# Feasibility of a novel, synthetic, self-assembling peptide for suture-line haemostasis in cardiac surgery

**DOI:** 10.1186/s13019-018-0745-2

**Published:** 2018-06-15

**Authors:** Suresh Giritharan, Kareem Salhiyyah, Geoffrey M. Tsang, Sunil K. Ohri

**Affiliations:** 1 Wessex Cardiac Centre, University Hospitals Southampton, Tremona Road, Southampton, SO16 6YD UK; 2 Southampton, UK

**Keywords:** Cardiac surgery, Bleeding, Haemostasis, Haemostatic agents, Blood loss, Blood products, Transfusion

## Abstract

**Backgroud:**

To assess the feasibility and efficacy of PuraStat®, a novel haemostatic agent, in achieving suture line haemostasis in a wide range of cardiac surgical procedures and surgery of the thoracic aorta.

**Methods:**

A prospective, non-randomised study was conducted at our institution. Operative data on fifty consecutive patients undergoing cardiac surgery where PuraStat® was utilised in cases of intraoperative suture line bleeding was prospectively collected. Questionnaires encompassing multiple aspects of the ease of use and efficacy of PuraStat® were completed by ten surgeons (five consultants and five senior registrars) and analysed to gauge the performance of the product.

**Results:**

No major adverse cardiac events were reported in this cohort. Complications such as atrial fibrillation, pacemaker requirement and pleural effusions were comparable to the national average. Mean blood product use of packed red cells, platelets, fresh-frozen plasma (FFP) and cryoprecipitate was below the national average. There was one incidence of re-exploration, however this was due to pericardial constriction rather than bleeding. Analysis of questionnaire responses revealed that surgeons consistently rated PuraStat® highly (between a score of 7 and 10 in the various subcategories). The transparent nature or PuraStat® allowed unobscured visualisation of suture sites and possessed excellent qualities in terms of adherence to site of application. The application of PuraStat® did not interfere with the use of other haemostatic agents or manipulation of the suture site by the surgeon.

**Conclusion:**

PuraStat® is an easy-to-use and effective haemostatic agent in a wide range of cardiac and aortic surgical procedures.

## Background

Meticulous haemostasis remains a core principal in surgical practice, both for patient safety and for preserving the integrity of surgical repair [1]. The challenge of cardiac surgery involves operating on patients who are receiving single and dual antiplatelet or anticoagulant therapy, systemic heparinization and the effects of hypothermia on the clotting cascade [2]. The consequences of massive haemorrhage are well recognised and rare in modern practice as bleeding into the pericardial space can lead to haemodynamic compromise by cardiac tamponade.

**Fig. 1:**
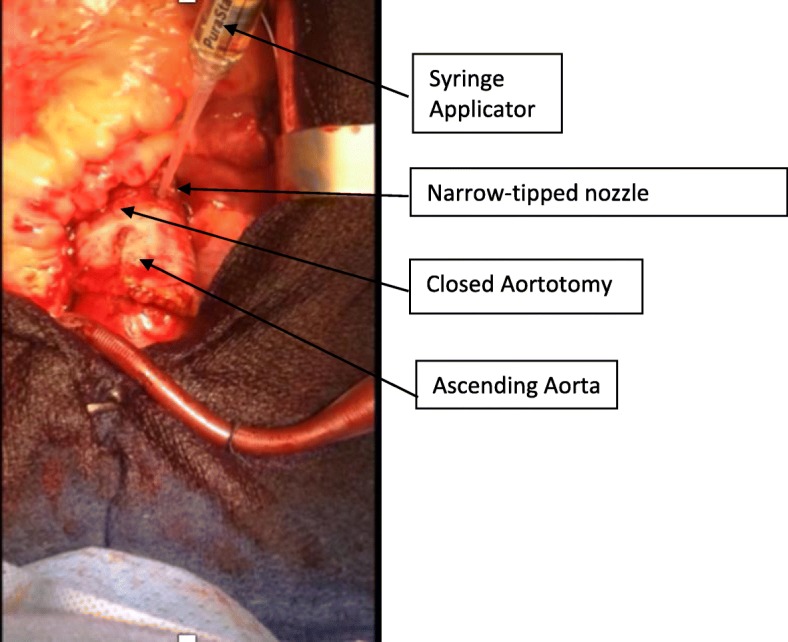
Application of PuraStat® via syringe applicator with a long, narrow nozzle over aortotomy suture line

**Fig. 2:**
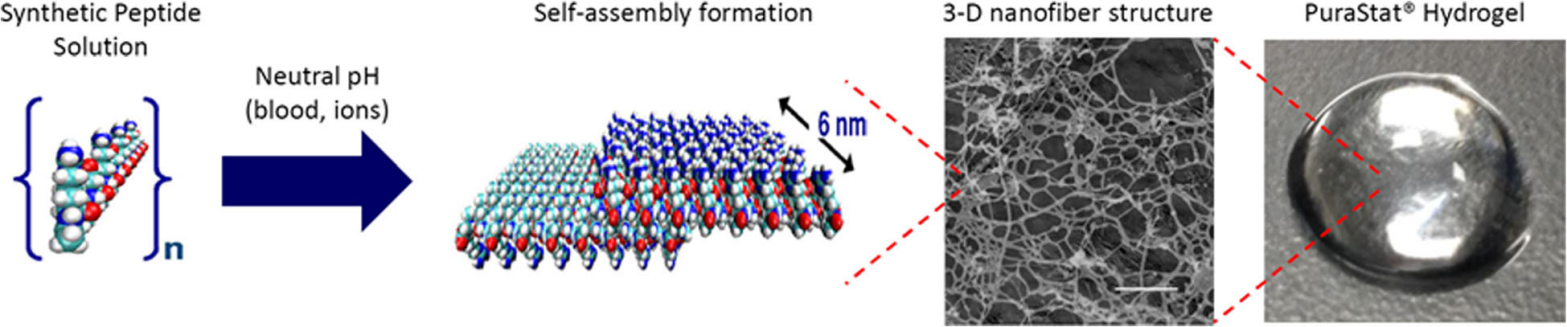
Contact between PuraStat® and physiological liquid such as blood causes the acidic peptide solution to approach neutral pH and be exposed to ions. This triggers self-assembly of ß-sheets and then nano-fibres within a hydrogel. The hydrogel rapidly covers the point of bleeding and provides a physical surface under which coagulation occurs to achieve haemostasis. (figure courtesy of 3-D Matrix)

The search for an ideal haemostatic adjunct to complement suturing of cardiac and aortic tissue and in fashioning anastomoses is ongoing, as the agent must possess a comprehensive catalogue of qualities such as ease of application, good adherence to site of use, cause minimal tissue reaction and prove robust in withstanding volume and pressure changes generated from the beating heart [3]. Currently there are many established haemostatic agents on the market with varying mechanisms of action, which attests to the fact that there is no one ideal agent (Figs. 1 and 2).

In this study we report our experience using PuraStat® (3-DMatrix Inc., Massachusetts) a novel synthetic haemostat. It relies upon nanotechnology with the self-assembling of constituent peptides, which is triggered by sodium ions within the blood to form a synthetic, non-biogenic, biocompatible, resorbable peptide hydrogel with no risk of transmissible spongiform encephalopathies [4]. It is CE-marked for haemostatic use in humans and is currently being used successfully as a standalone haemostatic agent in endoluminal gastrointestinal procedures, among other applications [5]. Our aim was to investigate the haemostatic efficacy of PuraStat® for cardiac and aortic anastomoses and suture lines.

## Methods

A prospective, non-randomised study was conducted at our institution between February 2017 and September 2017. A total of fifty patients undergoing cardiac surgery at the Wessex Cardiothoracic Centre (Southampton, UK) and The Spire Hospital (Southampton, UK) were recruited. The inclusion criteria encompassed all adult cardiac surgery cases where the intraoperative use of a haemostatic agent was deemed necessary. A comprehensive case-mix including coronary artery bypass surgery, aortic valve surgery, mitral valve surgery, surgery on the thoracic aorta and other concomitant procedures (e.g. atrial fibrillation ablation procedures) were included, encompassing elective, urgent and emergency presentations. Patients whom exhibited preoperative derangements in haematological and coagulation profiles, and baseline derangements in liver function were excluded. Baseline demographic data such as age, gender, BMI, hypertension, diabetes, hypercholesterolaemia, previous myocardial infarction (MI), left ventricular (LV) function, smoking status, asthma or chronic obstructive pulmonary disease (COPD), renal impairment, neurological impairment and peripheral vascular disease was collected from pre-admission clerking sheets. Additional data included the use of preoperative antiplatelet or anticoagulant therapy. Preoperative blood results for haemoglobin, platelet count, INR, APTR and fibrinogen were also noted.

Intraoperative data included details of the operation, cardiopulmonary bypass time, aortic cross-clamp time and site of use of PuraStat® and if additional haemostatic agents were required to achieve haemostasis. In all cases, the application of PuraStat® was done following reversal of systemic heparinization with Protamine Sulfate.

As the purpose of this study was to evaluate feasibility and efficacy, a questionnaire was developed with a view of gaining detailed observation and feedback on the use of PuraStat® in various scenarios. These questionnaires were given to the operating surgeon to complete following use of the product. The surgeons were asked to score the product on a scale of 1–10 for various factors. These were site of application, grade of bleeding, ease of preparation, time of preparation, ease of delivery to the sterile field, ease of use, delivery to the sterile field, application, conformation to irregular surfaces, compatibility with other haemostatic agents (when used), and reduction in haemostatic time. The questionnaire paid particular emphasis on the efficacy of PuraStat® alone in achieving haemostasis, and if not, what other haemostatic agent was used in conjunction with PuraStat®. Surgeons were also asked about their overall satisfaction with PuraStat®. A total of ten surgeons (five consultants and five senior registrars) provided feedback.

Chest drain output over the first 24 h was recorded along with the total use of blood products (packed red blood cells, platelets, fresh frozen plasma (FFP) and cryoprecipitate). Postoperative complications such as death, postoperative myocardial infarction, stroke, re-exploration for bleeding, atrial fibrillation, other arrhythmia, pleural effusion and reintubation were collated from a review of contemporaneously-completed inpatient notes. The results were then tabulated, with categorical variables expressed as a percentage of the total patient population and continuous variables expressed as the mean value with standard deviations. The mean rating for each criterion gauged in the questionnaire was calculated.

## Results

### Baseline demographics

Within the seven-month period, a total of fifty patients were recruited, and their baseline demographics are displayed below (Table 1) The mean age was 71.9 ± 10.4 years and the mean body mass index (BMI) was 28.7 ± 4.8 kg/m^2^. Thirty (60%) of participants were male and twenty (40%) were female. In terms of preoperative comorbidities, twenty-eight (56%) had hypertension (defined as patients receiving antihypertensive medications or having known but untreated hypertension [blood pressure > 140/90 mmHg]), eleven patients (22%) had diabetes (fasting glucose > 7 mmol/L), five patients had preoperative myocardial infarction (10%), three patients (6%) had chronic obstructive pulmonary disease (COPD) and four patients (8%) had peripheral vascular disease (PVD). Thirty-six (72%) of the participants had never smoked.

**Table 1:** Baseline Demographics of Patients

Variable	number (% of total)	mean	SD
Age		71.9	10.4
*Gender*			
Male	30 (60%)		
Female	20 (40%)		
BMI (kg/m^2^)		28.7	4.8
Hypertension	28 (56%)		
Diabetes	11 (22%)		
Myocardial Infarction?	5 (10%)		
Asthma/COPD	3 (6%)		
Peripheral Vascular Disease	5 (10%)		
Never smoked	36 (72%)		
*Left Ventricular Function*			
Good	45 (90%)		
Moderate	4 (8%)		
Poor	1 (2%)		
Preoperative Blood Results			
Haemoglobin		130.5	14.3
Platelets		240.2	71.6
INR		1.06	0.12
APTR		1.05	0.2
Fibrinogen		4.54	1.18

Values are total number of patients, n (% of n), mean and standard deviation (SD). BMI, body mass index; MI, myocardial infarction; COPD, chronic obstructive pulmonary disease; INR, international normalized ratio; APTR, activated partial thromboplastin time ratio

All patients in this cohort underwent preoperative transthoracic echocardiographic examination. Forty-five (90%) of patients had good left ventricular function, four patients (8%) had moderate left ventricular function and one patient (2%) had poor left ventricular function. Blood results taken prior to surgery demonstrated mean values for haemoglobin level (130.5 ± 14.3 g/dL), platelet count (240.2 ± 71.6 × 10e9/L), INR (1.05 ± 0.12), APTR (1.05 ± 0.2) and fibrinogen (4.54 ± 1.18 g/L).

### Type of operation and site of use

**Fig. 3:**
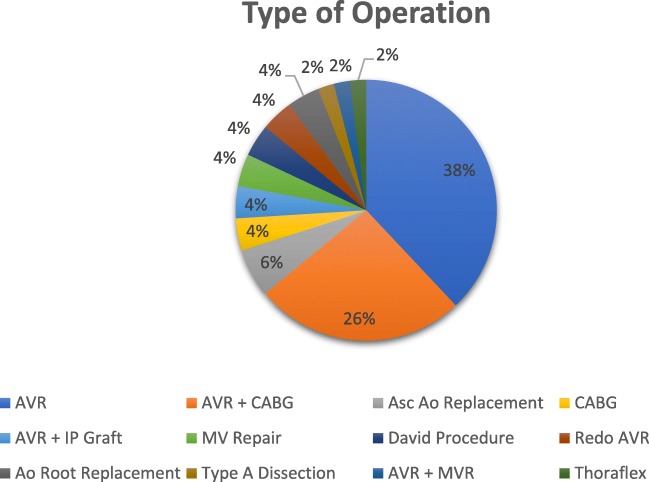
Type of Operation. AVR, aortic valve replacement; MV, mitral valve; CABG, coronary artery bypass grafts; Asc Ao, Ascending Aorta

**Fig. 4:**
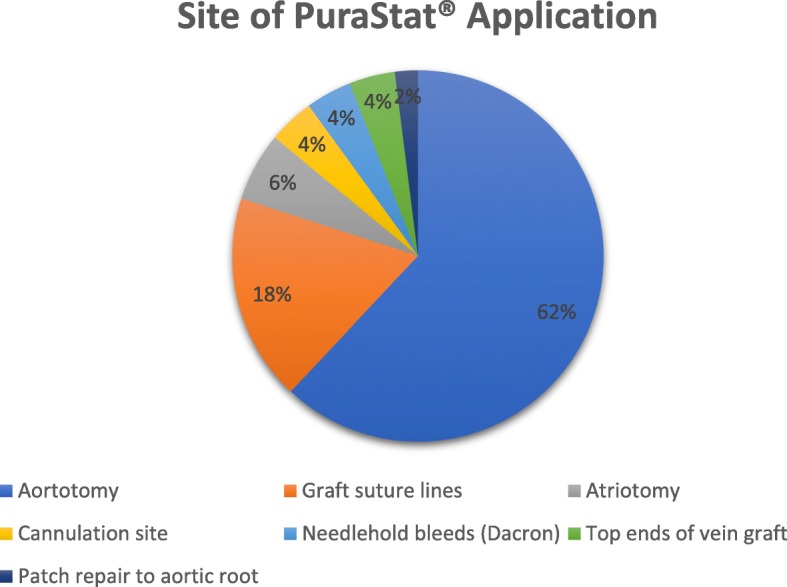
Site of Use of PuraStat®

Figures 3 and 4 illustrate the type of operations performed as well as the site of PuraStat® use. In all cases, the application of PuraStat® was done following reversal of systemic heparinization with Protamine Sulfate. Thirty eight percent of patients underwent aortic valve replacement, 26% underwent aortic valve replacement and concomitant coronary artery bypass surgery and 6% of patients had replacement of the ascending aorta with a prosthetic graft. Four percent of patients each had aortic valve replacement with concomitant interpositional graft placement, isolated coronary artery bypass graft surgery, mitral valve repair, valve-sparing aortic root replacement (David Procedure), redo aortic valve replacement and aortic root replacement. Two percent of patients each had emergency repair of a Type A dissection, concurrent aortic and mitral valve replacement and aortic hemiarch replacement with a Thoraflex graft. PuraStat® was most frequently used at the site of aortotomy closure (62%), followed by haemostasis of graft suture lines (18%). It was used to aid haemostasis on closure of the site in 6% of cases, and for 4% of cases each was applied to aortic and right atrial cannulation sites, to needle hole bleeds from prosthetic grafts and to the top ends of vein grafts during CABG surgery. In one case (2%) it was used to aid the fashioning of a pericardial patch which was implanted onto the aortic root.

### Total blood product use and 24-h blood lost via intercostal drain output

Table 2 details the mean number of units for blood products such as packed red blood cells (blood), platelets, fresh frozen plasma (FFP) and cryoprecipitate. Additionally, the average number of units of Prothrombin Complex Concentrate (Octaplex®) is included. These results were compared to those of the 2011 Audit of Blood Transfusion in Adult Cardiac Surgery (National Comparative Audit of Blood Transfusion) [6]. Mean values for units of packed redcells (1.45 ± 1.99), platelets (1.22 ± 1.07), fresh frozen plasma (0.94 ± 1.36) and cryoprecipitate (1.33 ± 1.73) were lower than the national average. Mean units of Octaplex use was 1.19 ± 1.54. The percentage reduction of use of blood products in this cohort as compared to the national average was 51.34% for packed red cells, 34.05% for platelets, 52.5% for fresh-frozen plasma and 16.35% for cryoprecipitate.

**Table 2:** Total blood product use compared to the national average

Blood Products Used	Mean units, n	SD	UK Average (CABG)	reduction (%)
Packed Red Cells	1.45	1.99	2.98	51.34%
Platelets	1.22	1.07	1.85	34.05%
FFP	0.94	1.36	1.98	52.50%
Cryoprecipitate	1.33	1.73	1.59	16.35%
Octaplex	1.19	1.54	n/a	n/a

Table 3 highlights the mean blood loss via intercostal drain output for the first 24 h following surgery and the total cell-salvaged volume transfused following surgery. All patients were infused with the total volume of cell-salvaged blood from cardiopulmonary bypass within the first 24 h following surgery.

**Table 3:** Mean cell-salvaged blood infused and 24-h chest drain output

Cell-salvaged blood and Chest Drain Output	Mean Volume, ml	SD
Cell-salvaged blood infused	668.73	280.8
Chest Drain output (24 h)	362.07	287.06

### Questionnaire results

Table 4 lists the mean scores given by ten surgeons on their experience when using PuraStat®. Every surgeon surveyed has had considerable experience performing the operations in which they used the product, has had the opportunity to use the product more than once and has had experience using similar products from other manufacturers. All theatre scrub staff received training on the product prior to use. PuraStat® scored very highly on pre-application factors such as preparation of the product and dispensation from the applicator. A mean score of 9.7 was assigned for ease of application to the target site. Scores of 8.5, 8.3 and 8.9 respectively were assigned for application to hard-to-reach surfaces, conformation to irregular surfaces and ease of removal (of excess material) from the surgical field. Surgeons found PuraStat® worked well with other agents, reporting no issues such as smudging, sticking or impaired adherence to the target site when used with Fibrillar®(classified as a haemostat) or BioGlue®(classified as a sealant/glue). It was thought to be valuable in reducing haemostasis time (mean score = 7.5), and subsequently operating time (mean score = 7.3), and in 84% of the cases was adequate in achieving haemostasis without the aid of other haemostatic agents (8% used concurrently with Fibrillar®, 6% used with both Fibrillar® and BioGlue® and 2% used concurrently with BioGlue®).

**Table 4:** Mean user evaluation scores on the feasibility and efficacy of PuraStat®

Surgeons’ Rating of PuraStat®	Average score (1–10)
Ease and efficiency of preparation prior to use	10
Easy to dispense from applicator	10
Ease of application to site of bleed	9.7
Application to hard-to-reach surfaces	8.5
Conformation to irregular surfaces	8.3
Ease of removal of excess PuraStat® from field	8.9
Concomitant use with other haemostatic agents	9.4
Valuable in reducing haemostasis time?	7.5
Potential in reduction of operating time?	7.3
PuraStat® alone enough to achieve haemostasis?	**YES = 84%**
	No = 16%
Other products used concomitantly	None = 84%
	Fibrillar® = 8%
	BioGlue + Floseal = 6%
	BioGlue = 2%

### Postoperative complications

Table 5 lists the inpatient postoperative complications for this cohort of patients. No deaths, cerebrovascular accidents (CVA) or myocardial infarctions (MI) were reported. Fourteen patients (28%) experienced new postoperative atrial fibrillation while ten patients (20%) had pleural effusions confirmed on a chest radiograph. None of the patients were re-explored for bleeding; one patient required surgical re-exploration due to a presentation mimicking cardiac tamponade, however this turned out to be pericardial constriction causing a low cardiac output status. No bleeding was reported from this event.

**Table 5:** Postoperative Complications

Postoperative Complications	Number
AF	14 (28%)
Pleural effusion	10 (20%)
CVA	0
Re-exploration for bleeding	0
MI	0
Other arrhythmias	3
Death	0

## Discussion

A major challenge when confronted with small-volume, persistent oozing from vascular structures is the limited role of suturing, as every additional bite taken will result in more needle-hole bleeding points. The aforementioned insults to the intrinsic physiological clotting mechanisms from antiplatelet therapy, systemic heparinization and cooling further exacerbate this problem [7, 8]. The current selection of topical haemostatic agents on the market have demonstrated satisfactory haemostatic properties, but has the potential pitfall of enabling transmission of viral and prion diseases (in the case of human blood component-derived substances), and instigating a systemic inflammatory response syndrome to animal-derived peptide-based products [9, 10]. The risk of such complications is negated by the use of a completely synthetic agent like PuraStat®.

In this consecutive case series surgeons reported several distinct qualities of PuraStat® which were felt to be advantageous. The transparent nature provided surgeons with the novel opportunity to maintain visualisation of the suture line after application of the haemostat. This allowed the opportunity for surveillance of the operative field for slow accumulation of haematoma sandwiched between the vessel and the haemostat layer during the course of the remainder of the operation. A threatened or suboptimal suture repair can then be revised if needed, potentially preventing the need for postoperative re-exploration. The method of delivery and application as a viscous gel rather than a spray applicator was an essential feature when operating within a tight surgical field, enhancing precision (i.e. application over the aortotomy with multiple vein grafts invading the field of view).

The viscosity profile of PuraStat® which demonstrates a more cohesive rather than adhesive characteristic, makes it ideal for concomitant use with other haemostatic materials (in this case, BioGlue® and Fibrillar®). Cross-compatibility with such products is essential, especially in complex aortic procedures in which deep hypothermic circulatory arrest (DHCA) is utilised as the dysregulation of coagulative mechanisms is even more pronounced.

### Limitations

Our primary aim in this study was to determine feasibility of PuraStat® in aiding haemostasis in cardiac surgery. By evaluating the use this product in fifty consecutive cases, we have established that in a wide variety of surgical scenarios, PuraStat® is shown to be effective in achieving haemostasis. Patients in this study were not stratified according to preoperative risk factors for general morbidity and mortality from cardiac surgery. Neither were they stratified for their individual risks of bleeding and poor tissue healing (i.e. preoperative antiplatelet therapy, type 2 diabetes, corticosteroid use, congestive heart failure and chronic renal failure). The type, length and urgency of the operation would also influence major adverse cardiac events and postoperative bleeding. We propose a follow-up study where patients are propensity-score matched for these criteria [11].

The variation of surgical practice and experience between the ten surgeons resulted in variations in the strategy of application of PuraStat® (i.e. precise location of application and quantity) as well as judgement on how quickly haemostasis was achieved. The point at which different surgeons deemed the product insufficient for haemostasis (and subsequently a different product or change in operative strategy was employed) was not recorded. Previous trials of the efficacy of haemostatic agents have assessed efficacy by standardising the anatomical point of use and time window in achieving bleeding control. Such stringent criteria would provide valuable objective, quantitative data.

## Conclusion

Our single-centre, qualitative evaluation found PuraStat® to be a feasible, safe and effective haemostatic agent in a wide range of cardiac and aortic surgical procedures. Specific qualities in terms of the transparent nature and viscosity profile of the product was found to be novel and valuable in our surgical practice. A further randomised-controlled study where patients are propensity-matched for baseline demographics, operative urgency, bleeding risk, tissue integrity factors and operation type is warranted to quantitatively evaluate the performance of PuraStat® in various specific circumstances.

## Abbreviations

AF: Atrial Fibrillation
Ao Root: Aortic Root
APTR: Activated Partial Thromboplastin Time Ratio
Asc Ao: Ascending Aorta
AVR: Aortic Valve Replacement
BMI: Body Mass Index
CABG: Coronary Artery Bypass Graft
COPD: Chronic Obstructive Pulmonary Disease
CVA: Cerebrovascular Accident
DHCA: Deep Hypothermic Circulatory Arrest
FFP: Fresh Frozen Plasma
INR: International Normalised Ratio
IP: Interposition
LV: Left Ventricle
MI: Myocardial Infarction
MV: Mitral Valve
MVR: Mitral Valve Replacement
PVD: Peripheral Vascular Disease
RA: Right Atrium
